# Determination of ABO Blood Groups and Rh Typing from Dry Salivary Samples

**DOI:** 10.5005/jp-journals-10005-1493

**Published:** 2018-04-01

**Authors:** Priyam R Velani, Preetam Shah, Laxmi Lakade

**Affiliations:** 1 Postgraduate Student, Department of Pedodontics, Bharati Vidyapeeth Deemed University Dental College and Hospital, Pune, Maharashtra India; 2 Professor, Department of Pedodontics, Bharati Vidyapeeth Deemed University Dental College and Hospital, Pune, Maharashtra India; 3 Associate Professor, Department of Pedodontics, Bharati Vidyapeeth Deemed University Dental College and Hospital, Pune, Maharashtra India

**Keywords:** ABO blood group, Absorption-inhibition method, Dried salivary samples, Rh typing.

## Abstract

**Introduction:**

A unique blood group is a characteristic every individual possesses. Blood group antigens like A, B, D, H, etc., are found to be present on the cell surfaces of red blood cells (RBCs). Besides blood, these are also secreted in various body secretions like semen, sweat, amniotic fluid, and saliva. Blood grouping has several applications in forensic sciences and is also a major part of routine medical investigations. Presence of these antigens in saliva is dependent on the secretor status of an individual. Saliva samples at the crime scene are very crucial, as they help in deoxyribonucleic acid typing, sex determination, bite mark analysis, and blood grouping. Dried salivary samples are often obtained in more number of cases as compared with the wet form, due to the variable time lapse between the occurrence of the crime and the start of the investigation. Blood grouping from these samples proves to be very efficient. Thus, the present study aims at evaluating the accuracy of ABO blood group determination and Rh typing from dried salivary samples. Also, the study would establish the use of saliva as a noninvasive technique in routine blood examinations, especially in children who have needle phobia.

**Materials and methods:**

Blood grouping and Rh typing were performed on the dry salivary samples obtained from the 47 subjects using the absorption-inhibition technique. This was then compared with the results obtained using extraction socket blood and evaluated.

**Results:**

The present study showed a 100% positive correlation for ABO blood grouping, but a mere 14.81% positive correlation for Rh typing between the dried salivary samples and the extraction socket blood.

**Conclusion:**

Dried salivary samples can thus be put to immense use in several areas of forensic investigations. It could also help in developing alternate methods for routine blood investigations in children and adults.

**How to cite this article:** Velani PR, Shah P, Lakade L. Determination of ABO Blood Groups and Rh Typing from Dry Salivary Samples. Int J Clin Pediatr Dent 2018;11(2):100-104.

## INTRODUCTION

Forensics in the recent past has gained a lot of importance and coverage. The ever-challenging crime scene investigations are greatly being benefitted with the use of ultramodern technologies and inventions. The main aim of every investigation is to establish the identity of the culprit from the various suspects. Forensic dentists play a major role in several such investigations and prove to be a major aid in solving crimes. Forensic odontology is a major branch of forensic sciences. Blood at the crime scene proves to be a huge benefit for the investigator. However, in some medico-legal cases like those involving child abuse, rape, robbery, and murders, blood may be absent at the scene, but other biological evidences may be present and obtainable. These mainly include fingerprints, hair samples, semen, and saliva. In many such cases, saliva may be obtained in a dry or wet form from human bite marks, cigarette ends, envelope flap openings, dental appliances, food items, plastic casings, etc. Dried salivary samples are often obtained in more number of cases as compared with the wet form, due to the variable time lapse between the occurrence of the crime and the start of the investigation. Thus, the dried salivary samples which are more significant can be put to great use in finding out the culprit.

The blood group of an individual, once established at birth, remains constant throughout one’s life. Hence, blood groups can be successfully used for establishing the identity of an individual and in narrowing down the suspect pool. Blood groups are basically antigenic determinants present on the surface of RBCs. Landsteiner was the first to describe the ABO grouping of blood. Following this, several other blood group types like Rh, P, Kell, MN, Lewis, Kidd, Diego, etc., were put forth. However, only the ABO and Rh groups are in practice for clinical and forensic use. Other blood groups are of less importance because the antigens are weak and the corresponding antibodies are not normally present and appear only after multiple transfusions.^1^

Besides blood, the blood group antigens are also secreted in various other body secretions, such as saliva, semen, gastric juice, nasal secretions, vaginal secretions, sweat, tear, urine, etc., from which blood groups can be determined.^2^ However, the presence of blood group substances in various body secretions depends on whether the individual is a secretor or a nonsecretor, which can be determined by the presence of Lewis antigen.^3^ In some medico-legal cases like those involving child abuse, rape, robbery, and murders, blood may be absent at the scene, but saliva in some form may be procured. This greatly helps in determining the blood group of a victim or a suspect. In such cases, saliva may be obtained in dry forms from bite marks, cigarette ends, envelope flaps, dental appliances, etc.^4^

**Fig. 1:**
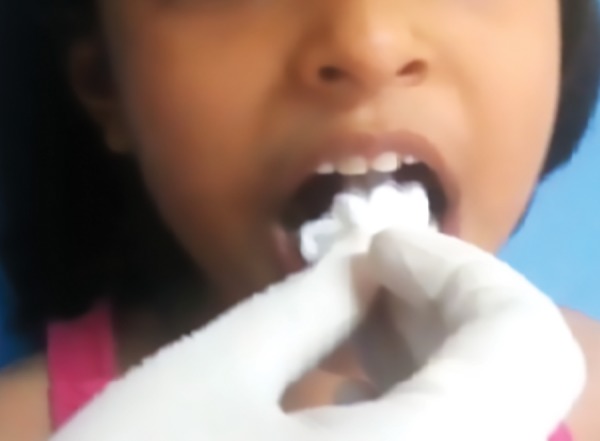
Collection of saliva sample using gauze piece

**Fig. 2:**
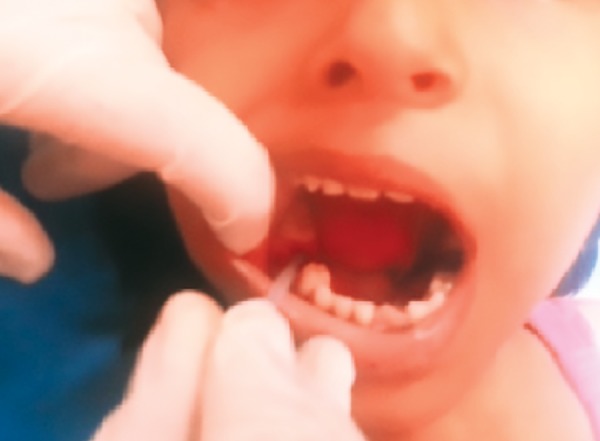
Collection of blood using disposable pipette

In recent years, many new techniques have been introduced and many investigations have shown up to 100% accuracy in detecting ABO blood groups from saliva. There are mainly two methods for detecting ABO blood group substances from saliva.^5^

1. Absorption-inhibition method
2. Absorption-elution method

The absorption-inhibition method is the easier and simpler of the two.

Moreover, establishing the use of saliva in blood grouping could also further help establish the use of saliva as a noninvasive technique in routine blood examination, especially in children Thus, the present study aims at evaluating the accuracy of ABO blood group determination and Rh typing from dried salivary samples.

## MATERIALS AND METHODS

The study was conducted in the Department of Pediatric and Preventive Dentistry, Bharati Vidyapeeth Deemed University Dental College and Hospital, Pune, after approval of the ethical committee. Healthy children between the age group of 6 and 15 years whose teeth were indicated for extraction either due to physiologic mobility or for orthodontic purposes were chosen for the study.

Children with systemic diseases and those with mental disabilities were excluded from the study. An informed consent was obtained from the parents of all the subjects participating in the study.

### Collection of Samples

**Fig. 3:**
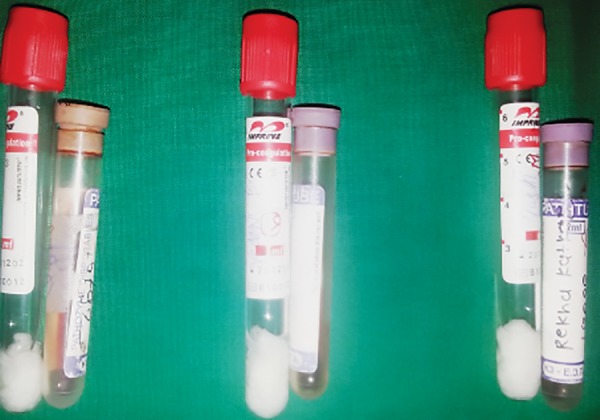
Saliva and blood samples in vacuum collection tubes

Before the extraction procedure, a small piece of gauze was given to each child, who was then asked to wet it with his/her own saliva (Fig. 1). Soon thereafter, the saliva sample was collected, and the extraction was carried out. A volume of 1 mL of socket blood was collected using a dropper inside an ethylenediaminetetraacetic acid bulb (Fig. 2). After the collection of these samples (Fig. 3), the wet gauze pieces were allowed to dry at room temperature. Once they dried up, they were taken in a collection tube, diluted with 3 to 4 mL of distilled water and allowed to rest overnight. The blood samples were immediately taken to the lab for blood grouping.

Blood grouping and Rh typing from socket blood were done using the slide agglutination method. Before carrying out blood grouping and Rh typing from the dried salivary samples, the presence of amylase activity was checked for each dried sample using commercially available alpha amylase reagents. Only, once the positive amylase activity was determined, were the samples considered fit for blood grouping and Rh typing.

**Fig. 4:**
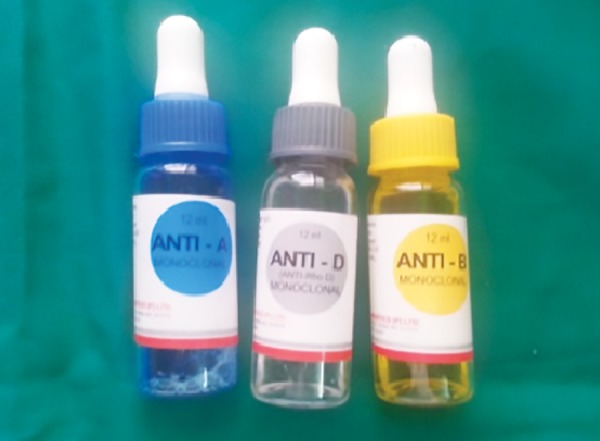
Anti A, Anti D, and Anti B antisera

To determine if a person is a secretor, the principle of *Agglutination Inhibition* is utilized. It works through two processes.

1. Antibody Neutralization: Saliva is mixed with commercial antisera and allowed to incubate briefly. If the patient is a secretor, the blood group antigens in the saliva will react with and neutralize the antibodies in the commercial antiserum. It is necessary, however, to dilute the commercial antiserum so that its antibody titer more closely matches the antigen level in the saliva.
2. Agglutination Inhibition: When commercial RBCs of the appropriate blood group are added to the test mixture, there should be no free antibody to agglutinate them if the patient is a secretor, because the antibodies have already reacted with the blood group antigens in the saliva. The reaction will be negative for agglutination, but is interpreted as positive for secretor status.

If the patient is a nonsecretor, there will be no blood group antigens in the saliva; the antibodies in the antise-rum will not be neutralized and will be free to react when the test cells are added. Therefore, agglutination is a negative test for secretor status. Hence, for our samples, the presence of agglutination means a negative test, and the absence of agglutination is interpreted as a positive result.

### Procedure

**Fig. 5:**
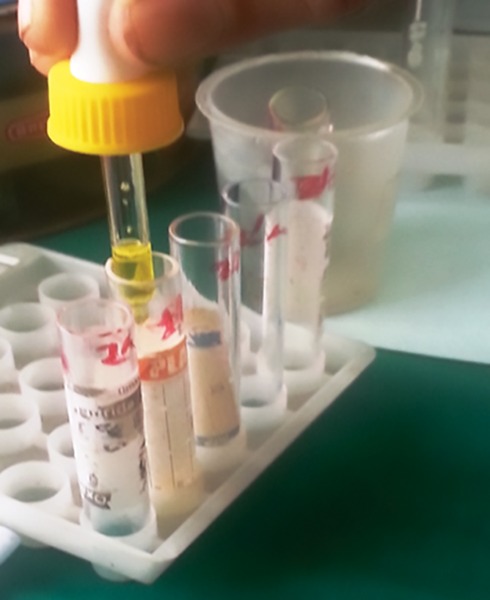
Adding of antisera to the respective test tubes

**Fig. 6:**
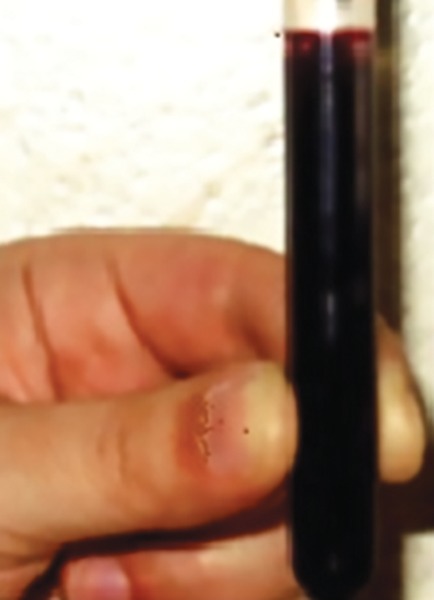
Blood containing tube for collection of fresh RBCs

**Fig. 7:**
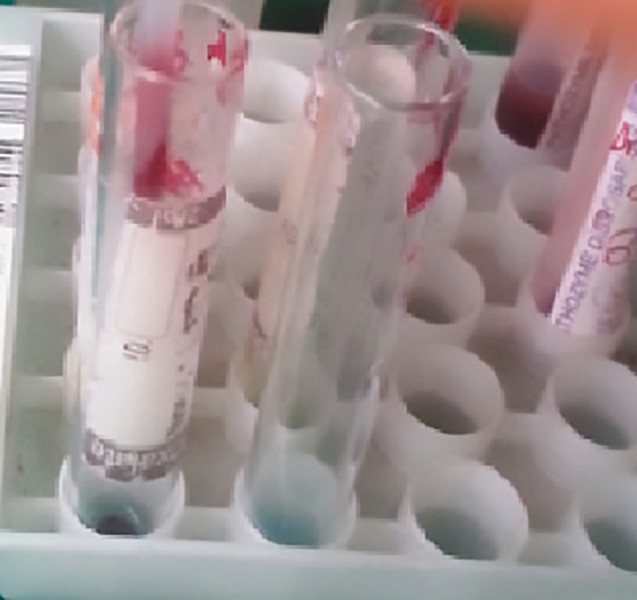
Checking for the presence or absence of agglutination

In order to denature the saliva, the diluted salivary sample is placed in a boiling water bath for 10 minutes. This inactivates enzymes that might otherwise destroy blood group substances. Allow to cool briefly and then transfer it to a 12 × 75 mm tube. Centrifuge for at least 5 minutes. The denatured saliva after cooling was taken in four test tubes. A, B, D, and H antisera in the dilution of 1:10 was added to each test tube, and the test tubes were respectively labeled (Figs 4 and 5). A singe drop from the salivary sample was added to both the test tubes, thoroughly shaken and incubated at 37° for 10 minutes. After 10 minutes, a single drop of freshly prepared pooled RBC of known group (Fig. 6) was added to the respective test tube, shaken well, and further incubated for 15 minutes at 37°. The samples were then checked for the presence or absence of agglutination (Fig. 7). All the results were compared with that obtained from socket blood and the results were statistically analyzed.

### Interpretation

- Agglutination in all of the patient test tubes indicates a negative result for secretor status.
- If any one of the patient test tubes is not agglutinated, this indicates a positive test for secretor status, and the tube showing the nonagglutination should indicate the ABO and Rh type.

## RESULTS

In this study, 47 subjects were studied for the determination of ABO blood groups and Rh typing from dried salivary samples and correlating the outcome with the blood samples obtained from the extraction sockets. Out of these, three samples were discarded owing to contamination of the blood.

The samples showed that 100% of the subjects were secretors. Moreover, ABO Blood groups determined from the dried salivary samples showed a 100% correlation with the blood groups determined from the extraction socket blood. Among the samples tested for ABO blood grouping, 7 samples showed blood group A, 7 showed blood group B, 16 showed blood group AB, and 14 showed blood group O (Table 1). For positive Rh typing, however, only a meager 14.81% positive correlation was obtained between the dried salivary samples and the extraction socket blood, wherein only four dried salivary samples tested positive as compared with 27 extraction socket blood samples (Table 2).

## DISCUSSION

Blood group of an individual, once established at birth, remains constant throughout one’s life. Hence, blood groups can be successfully used for establishing the identity of an individual and in narrowing down the suspect pool. Indirect blood grouping from saliva has been very important in catching criminals in many a crime scene. Apart from forensics, it has several other applications too.

Pawan Motghare et al^4^ in their study used the absorption-inhibition method for blood grouping and found around 83% secretors among the subjects. ABO blood grouping from saliva yielded around 85% positive correlation to the blood samples. In a study conducted by Emberibe et al,^6^ they studied 176 subjects for the presence of ABH antigen in saliva using the absorption-inhibition method and their results showed 84.90% secretors. The present study showed a higher percentage of secretors as well as a 100% correlation between the ABO blood grouping from dried salivary samples and extraction socket blood.

The literature available on the presence of Rh antigen in body secretions is very scanty and inconclusive. Wiener and Forer,^7^ claimed that saliva is free of Rh substance. Levin and Ketzin,^8^ also failed to prove otherwise. However, Boorman and Dodd^9^ were the first to have claimed the demonstration of a small amount of Rh substance in Rh-positive individuals. In a series of 51 Rh-positive subjects, they reported evidence of secretion in 37 instances. They observed that the concentration of the Rh-specific substance in saliva is less as compared with the tissues.^10^ In the present study as well, Rh activity did not yield significant positive results.

Since, there is a lack of significant research wherein both ABO blood grouping and Rh typing have been attempted together from dried salivary sample, the present study was undertaken to co-relate the presence of A, B, H, and D antigens in the dried salivary samples with that obtained from the blood. There are several other applications for blood grouping from saliva. If a suitable and consistent method can be established, it could further propagate the use of saliva as a noninvasive technique in routine blood examinations, especially in children who have needle phobia. It could also prove to be an adjunct in cases of hematologic malignancies wherein an alteration of ABH antigens has been noted.^11^ Moreover, many studies have correlated the secretor status and many systemic illnesses like ankylosing spondylitis, peptic ulcer, ovarian cysts, and even squamous cell carcinoma.^12^ Thus, dried salivary samples proved to be a reliable source for ABO blood grouping; however, for the detection of D antigen, a more technique-sensitive approach needs to be developed.

**Table 1:** **Table 1:** Correlation of ABO blood grouping between dried salivary samples and extraction socket blood

*Blood group type*		*Dried salivary sample (number of samples)*		*Extraction socket blood (number of samples)*		*Dried salivary samples (%)*		*Extraction socket blood (%)*	
Blood group A		7		7		15.9		15.9	
Blood group B		7		7		15.9		15.9	
Blood group AB		16		16		36.3		36.3	
Blood group O		14		14		31.8		31.8	

**Table 2:** **Table 2:** Correlation of Rh typing between dried salivary samples and extraction socket blood

*Rh typing*		*Dried salivary sample (number of samples)*		*Extraction socket blood (number of samples)*		*Dried salivary samples (%)*		*Extraction socket blood (%)*	
Positive Rh activity		4		27		9.09		61.3	
Negative Rh activity		40		17		90.91		38.7	

## CONCLUSION

ABO blood grouping from dried salivary samples can thus be put to immense use in several areas of forensic investigations. It could also help in developing alternate methods for routine blood investigations in children and adults. For establishing Rh typing from saliva, a more sensitive approach needs to be devised and put to use.
